# Determination of the reference interval for urinary klotho to creatinine ratio of healthy dogs

**DOI:** 10.3389/fvets.2024.1423390

**Published:** 2024-07-23

**Authors:** Nikola Marečáková, Jana Kačírová, Csilla Tóthová, Aladár Maďari, Marián Maďar, Jana Farbáková, Slavomír Horňák

**Affiliations:** ^1^Small Animal Clinic, University of Veterinary Medicine and Pharmacy in Košice, Košice, Slovakia; ^2^Institute of Plant Genetics and Biotechnology, Plant Science and Biodiversity Centre, Slovak Academy of Sciences, Nitra, Slovakia; ^3^Clinic of Ruminants, University of Veterinary Medicine and Pharmacy in Košice, Košice, Slovakia; ^4^Department of Microbiology and Immunology, University of Veterinary Medicine and Pharmacy in Košice, Košice, Slovakia

**Keywords:** klotho, chronic kidney disease, urine analysis, biomarker, dog

## Abstract

For several years, alpha klotho has been considered as a candidate biomarker in chronic kidney disease (CKD), progression of CKD and CKD mineral bone disorders (CKD-MBD). The evidence on the relationship between klotho and kidney function is controversial in some areas. The aim of the study was to identify the influence of age, sex and breed on urinary alpha klotho, values in the early stages of CKD within the studied population and determine a reference interval in a group of healthy dogs. Significantly higher values were measured in older dogs over 6 years old (*p* = 0.026, *p* = 0.0007) and in the breed German Shepherd than Belgian Shepherd (*p* = 0.0401). On the basis of sex and in small breed dogs, no significant differences were noted. In dogs with CKD stage 2, alpha klotho values were significantly lower (*p* = 0.0135) than in healthy dogs. Within the studied population, a reference interval for urinary klotho to creatinine ratio (UrKl/Cr) was determined in the range of 3.94–23.55 pg/gCr. Since our findings show that alpha klotho is associated with older age, we assume that this may have influenced the results in the group of dogs with CKD stage 1 due to the presence of predominantly old dogs in this group. Future studies would be needed to consider age as a factor affecting urinary alpha klotho in dogs with CKD.

## 1 Introduction

Kidney disease is common in small animals and is often associated with a poor prognosis in the late stages (1). Chronic kidney disease (CKD) mainly affects older dogs. CKD is defined as structural and/or functional impairment of one or both kidneys that has been present for at least 3 months (2–4). CKD is a progressive, pathological disease that is the third most common cause of death in dogs (4). The prevalence of CKD in dogs varies widely (0.05–3.74%). Risk factors affecting the occurrence of the disease include advanced age, specific breeds, small body size and the presence of diseases such as periodontitis (5).

The International Renal Interest Society (IRIS) proposes a classification system for CKD into four stages based on the concentration of creatinine and symmetric dimethylarginine (SDMA) in the blood, and subclassifies based on the level of proteinuria and the presence of hypertension. However, until now there are no known biomarkers for the early diagnosis of CKD and disease progression that are sensitive, reliable, quantifiable and easily determined in routine practice (6).

Abnormalities of calcium and phosphorus homeostasis often occur in CKD, which have a negative impact on kidney function and survival. Approximately 76% of dogs with CKD develop complications of secondary renal hyperparathyroidism caused by hyperphosphatemia, which may be partly due to decreased urinary excretion, secondary to a decrease in GFR (7). In patients with CKD, a common comorbidity is chronic kidney disease-mineral bone disorder (CKD-MBT), accompanied by renal osteodystrophy and hyperphosphatemia (8, 9). The main hormones that regulate the manipulation of renal phosphates are parathyroid hormone (PTH), produced by the parathyroid gland, and fibroblast growth factor-23 (FGF-23), which is produced in bone by osteocytes and osteoblasts (10). FGF-23 levels increase before disturbances in mineral metabolism can be detected in people with CKD (11, 12). In dogs, FGF-23 also appears to predict the development of hyperphosphatemia and progression of CKD (13).

*In vivo* studies with genetically engineered FGF-23 mouse models provided compelling evidence for the phosphaturic activities of FGF-23 and provided explanations for the clinical symptoms observed in various diseases associated with abnormal FGF-23 regulation in humans (14–16). The FGF receptor-klotho complex ensures the action of FGF-23 to reduce NPT2a and NPT2c (sodium dependent phosphate co-transporters) expression in the proximal tubules in the kidneys (17). Alpha klotho is a single-pass transmembrane protein, the main source of which is the proximal and distal convoluted tubules in the kidneys, where its regulation also takes place, but it is also expressed in the parathyroid glands or in the choroid plexus (18–21). Downregulation of klotho receptors in the kidney limits FGF-23 signal transduction, resulting in increased PTH levels in CKD patients (22). In addition, soluble klotho directly controls phosphorus metabolism by regulating 1α-hydroxylase activity and secretion of PTH and FGF-23 in the kidney (16, 23).

Studies with human participants demonstrated that the concentration of alpha-klotho is reduced in both serum and urine in CKD patients (24, 25). Urinary levels of klotho in CKD patients have been shown to decrease significantly in very early IRIS Stage (1 and 2) CKD and was steadily reduced with the progression of disease (26, 27). Associations between soluble alpha-klotho levels with CKD and its clinical consequences, including CKD-MBD, have also been demonstrated in dogs (28).

Over the past decades, increasing evidence from various studies has shown that klotho, previously known as an anti-aging gene, is closely associated with the development and progression of CKD. Soluble alpha klotho may serve as an early and sensitive marker in the early stages of CKD due to the pathogenic mechanism of klotho deficiency and the decrease of urinary klotho in the early stages of CKD (22, 25). The aim of this study was to establish a reference interval for the urinary klotho to creatinine ratio in healthy dogs. We also assessed urinary alpha klotho as a possible early biomarker of CKD, its relationship to age, sex, and breed.

## 2 Materials and methods

### 2.1 Study population

A population of 96 dogs consisting of German Shepherds (GS), Belgian Shepherds (BS) and small breed dogs (SD) was included in the study. The SD group consisted of different breeds, mostly crossbreeds with a live weight of up to 15 kg (crossbreeds: *n* = 4, Yorkshire terrier: *n* = 1, Maltese dog: *n* = 1). The animals were handled in a humane manner. All applicable international, national and institutional guidelines for the care and use of animals were followed. Clinical examinations and sampling were performed with the informed consent of the dog owners. The animals were divided into groups according to the presence or absence of disease.

The criteria for the classification of healthy animals were:

- clinically healthy at the time of examination and sampling,
- without exposure to potentially nephrotoxic substances in the previous period of at least 6 months,
- hematology and biochemistry within reference intervals,
- urinary examination without pathological changes, UPC < 0.2,
- ultrasonographic examination without pathological findings,
- normotensive after BP examination,
- females were not pregnant or lactating.

After a complete clinical, laboratory, ultrasound and cardiological examination, 74 dogs (62 GS, 6 BS, and 6 SD) were included in the group of healthy animals (Table 1). The population consisted of 43 females and 31 males. GS were divided according to age into three groups: young individuals up to 2 years old, adults from 2 to 6 years old, and older individuals over 6 years old. Fifteen dogs were included in the group of animals with early stage of CKD based on the IRIS classification.^1^ After complete clinical, laboratory examinations and imaging results, CKD stage 1 (CKD1) was determined in seven dogs and CKD stage 2 (CKD2) in eight dogs. From both groups of CKD dogs, two patient were on a renal diet for a short time, without medical treatment.

**Table 1:** Distribution of dogs within the group of healthy animals.

	Total (*n*)	GS < 2 y. o.	GS 2–6 y. o.	GS > 6 y. o.	BS	SD
Female	43	12	19	6	1	5
Male	31	7	11	7	5	1

GS, German Shepherds; BS, Belgian Shepherds; SD, Small breed dogs; y.o., years old.

### 2.2 Collection and analysis of samples

Blood was collected from the *vena cephalica antebrachii* at least 12 h after the last feeding, and the blood samples were centrifuged (3,500 rpm, 10 min). Subsequently, the serum was subjected to a complete biochemical profile (AST, ALT, GGT, ALP, CK, Chol, Lip, Amyl, Crea, Urea, TBil, Glu, TP, Alb, P, Ca, K, Na, Cl) in the Cobas C 111 (Roche, Switzerland). SDMA level in serum was measured with the Catalyst One (IDEXX Laboratories, Westbrook, ME, USA). Hematological profile was analyzed in the ProCyte Dx (IDEXX Laboratories, Westbrook, ME, USA).

Urine with a volume of approximately 10 mL was collected by ultrasound-guided cystocentesis. After routine urinalysis, urine was centrifuged (2,500 rpm, 5 min), then samples were stored at 4°C and examined without freezing and thawing within 72 h. The concentration of alpha-klotho in urine was measured using a commercially available canine-specific sandwich enzyme-linked immunosorbent assay – Canine Soluble Alpha-Klotho (SAKL) ELISA kit (MyBioSource Inc., San Diego, CA, USA) following the manufacturer’s instructions. Briefly, standards and urine samples (50 μL) were applied to the ELISA plate and horseradish peroxidase-conjugate reagent (100 μL) was added. The microtiter plate was incubated for 60 min at 37°C, followed by manual washing. Then, chromogen solution A (50 μL) and chromogen solution B (50 μL) were added and incubated for 15 min at 37°C. Finally, stop solution (50 μL) was added. This quantitative assay is based on soluble alpha klotho antibody–antigen interactions and the intensity of the color reaction was evaluated spectrophotometrically on an automatic microplate reader Opsys MR (Dynex Technologies, USA) at a wavelength of 450 nm. Calibration was performed using a set standards from the kit at a range of the following concentrations: 1,000, 500, 250, 125, 62.5, 31.2 pg/mL. The sensitivity of the kit was 5.0 pg/mL. A precision test by applying coefficient of variation analysis was also conducted. The coefficient of variation obtained for SAKL was 7.46%. To minimize the effect of urine specific gravity, urine klotho to creatinine ratio (UrKl/Cr) was calculated (28).

### 2.3 Statistical analysis

Statistical analysis of the obtained data was performed using the GraphPad Prism 9.4.0 (GraphPad Software, San Diego, USA). An unpaired t-test with Welch’s correction at *p* < 0.05 or a one-way analysis of variance (ANOVA) with an additional Tukey’s test was used to determine statistical differences between dogs with CKD and healthy dogs, but also between dogs of different age, breed or sex. Interactions between the analyzed parameters of healthy dogs and dogs with CKD were evaluated using Pearson’s correlation analysis at *p* < 0.05.

The calculation method for reference interval and confidence interval with respect to the sample size, its descriptive statistics and the normality of the distribution was parametric with a logarithmic transformation. Outliers were identified and removed using the ROUTH method, *Q* = 1% in GraphPad Prism. Statistical tests for the occurrence of (potential) outliers and for normality were at the 5% significance level, the reference intervals were 95% and the confidence intervals for the limits were 90%.

## 3 Results

The results of laboratory analysis measuring the blood creatinine, urea, SDMA, and phosphorus and urine creatinine levels, urine soluble alpha klotho (UrKl) and urine klotho to creatinine ratio (UrKl/Cr) are presented in Table 2.

**Table 2:** Determined parameters of the serum and urine.

	Healthy dogs					Dogs with CKD	
	GS < 2 y. o.	GS 2–6 y. o.	GS > 6 y. o.	BS	SD	CKD1	CKD2
*n*	19	30	13	6	6	7	8
Crea (μmol/L) RI: 46–88	72.34 ± 15.54	83.67 ± 11.46	79.38 ± 15.13	81.83 ± 16.17	59.48 ± 11.06	102.96 ± 22.38	127.75 ± 17.88
Urea (mmol/L) RI: 3.97–8.05	5.35 ± 1.64	5.99 ± 1.41	5.50 ± 2.12	5.88 ± 1.68	5.94 ± 1.07	6.76 ± 2.43	8.74 ± 1.67
SDMA (mg/dL) RI <14	9.00 ± 1.80	8.25 ± 1.99	8.92 ± 2.15	7.67 ± 3.08	8.00 ± 1.79	8.57 ± 4.58	12.50 ± 6.00
P (mmol/L) RI: 0.9–1.91	1.95 ± 0.49	1.29 ± 0.17	1.19 ± 0.15	1.15 ± 0.47	1.51 ± 0.20	1.37 ± 0.16	1.31 ± 0.16
UCrea (mmol/L)	18.44 ± 8.98	19.45 ± 7.43	12.35 ± 7.25	18.48 ± 6.90	17.00 ± 4.89	20.04 ± 11.69	22.59 ± 6.90
UrKl (pg/mL)	198.43 ± 42.58	185.72 ± 54.85	207.13 ± 35.39	162.76 ± 34.81	191.92 ± 38.10	185.49 ± 28.82	199.13 ± 58.50
UrKl/Cr (pg/gCr)	12.66 ± 8.43	9.66 ± 4.28	21.21 ± 15.43	8.78 ± 3.45	10.75 ± 3.59	13.61 ± 14.09	8.46 ± 3.31

GS, German shepherds; BS, Belgian shepherds; SD, small breed dogs; y. o., years old; CKD1, chronic kidney disease stage 1; CKD2, chronic kidney disease stage 2; Crea, serum creatinine; SDMA, symmetric dimethyl arginine; UCrea, urinary creatinine; UrKl, urinary soluble alpha klotho; UrKl/Cr, urinary alpha klotho to creatinine ratio; RI, reference interval. Data are presented as mean ± SD.

**Figure 1:**
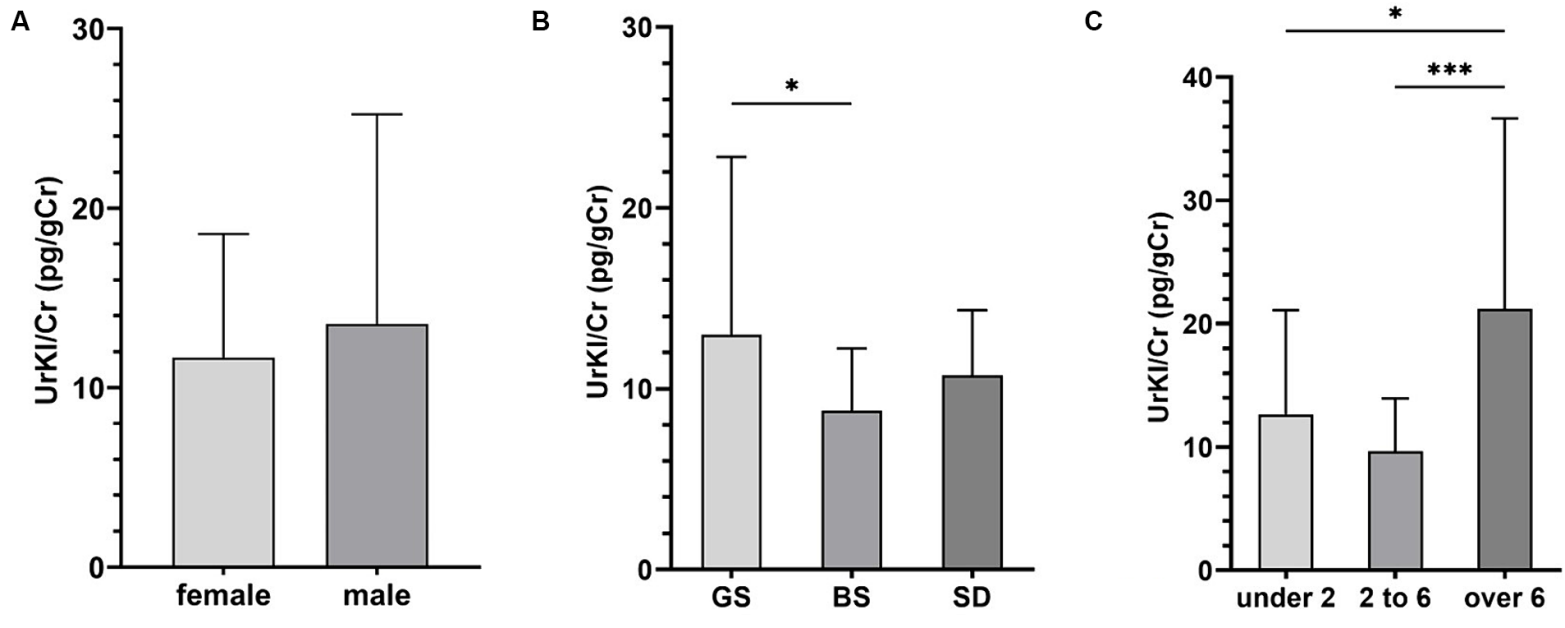
Concentration of UrKl/Cr based on sex **(A)**, breed **(B)**, and age – years **(C)**. Data are presented as mean ± SD. The asterisks represent a statistical significance, *p ≤ 0.05; **p ≤ 0.01; ***p ≤ 0.001.

In the group of healthy animals, the concentration of UrKl/Cr was higher in males, but the difference between males and females was not statistically significant (Figure 1A). Based on breed (Figure 1B), there were surprisingly significant differences between GS and BS (*p* = 0.0401), but non-significant differences between GS and SD (*p* = 0.2617). There was a statistically significant difference between different groups of GS based on age (*p* = 0.026, *p* = 0.0007). The highest values were measured in dogs older than 6 years (Figure 1C).

**Figure 2:**
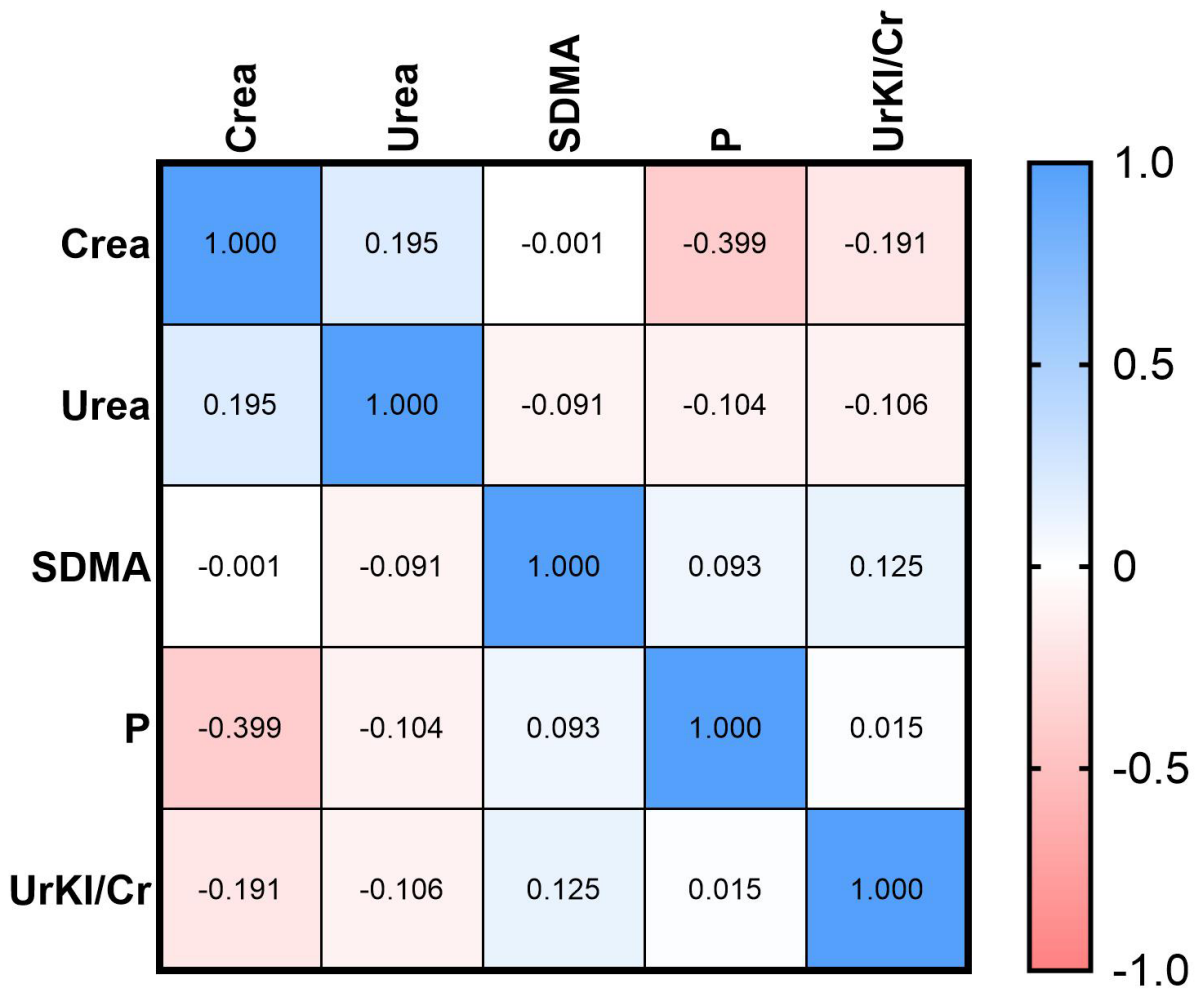
Relationship between UrKl/Cr and Crea, Urea, SDMA, and P in healthy dogs.

**Figure 3:**
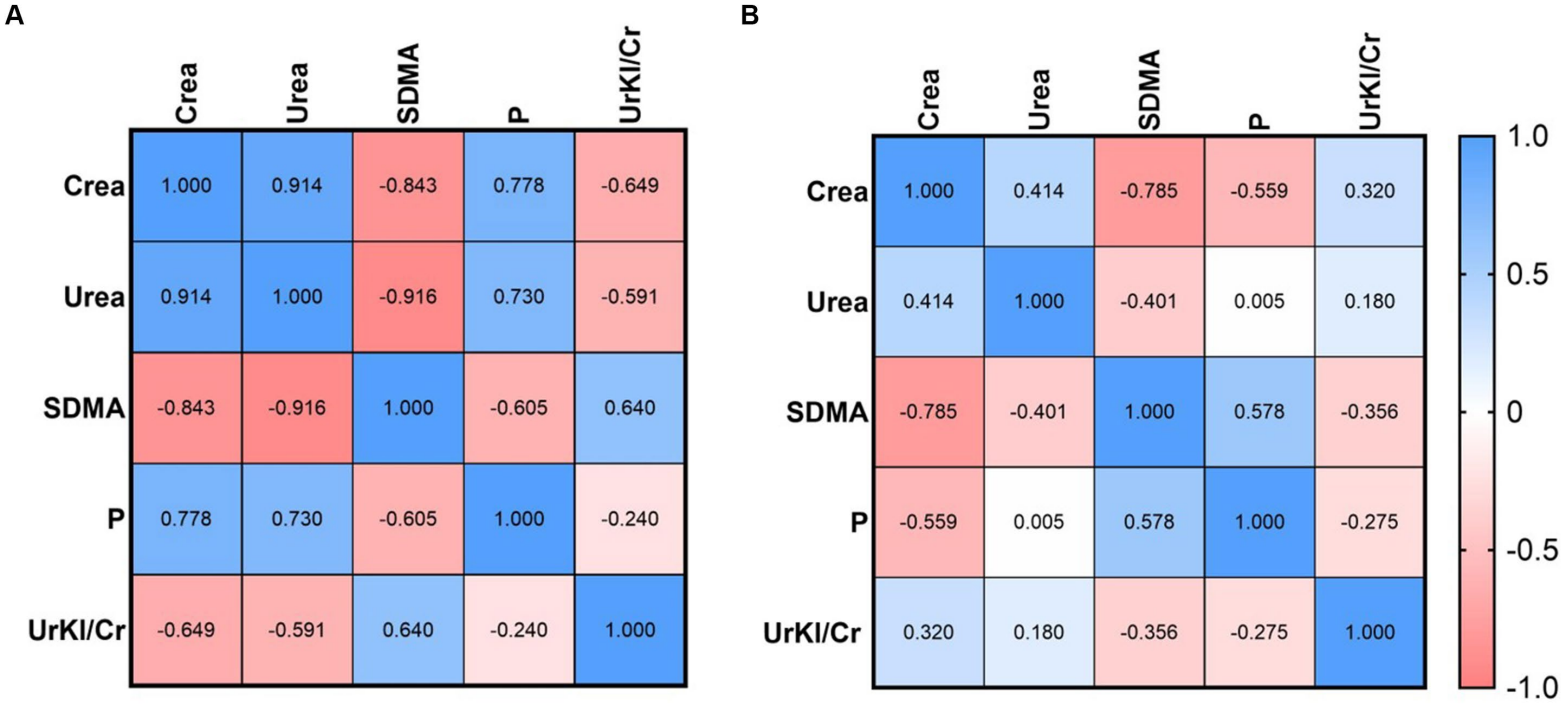
Relationship between UrKl/Cr and Crea, Urea, SDMA, and P in dogs with CKD1 **(A)** and CKD2 **(B)**.

Correlations between UrKl/Cr and other parameters were determined (Figures 2, 3A,B). There was no significant correlation between the concentration of UrKl/Cr and the concentration of P, SDMA, Crea or Urea in healthy dogs, but also in dogs with CKD. In healthy dogs, a significant negative correlation was found between P and Crea, while in dogs with CKD1, there was a significant positive correlation between these parameters. In dogs with CKD1, there was also a positive correlation between Crea and Urea and a negative correlation between SDMA and Urea and SDMA and Crea. In dogs with CKD2, there was only a negative correlation between SDMA and Crea.

**Figure 4:**
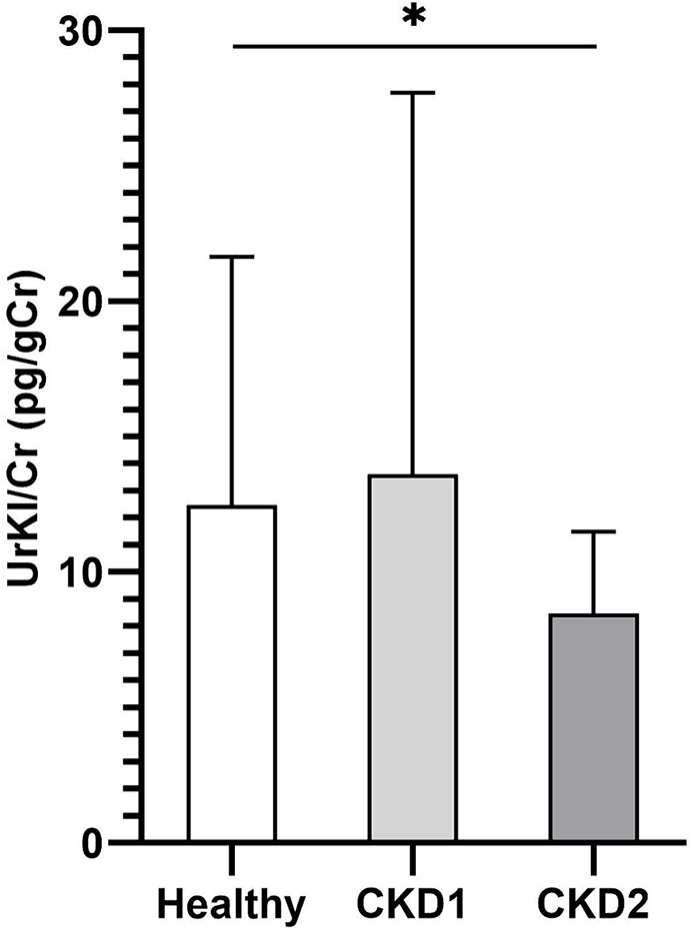
Concentration of UrKl/Cr in healthy dogs and dogs with CKD. Data are presented as mean ± SD. The asterisk represents a statistical significance, *p ≤ 0.05.

The mean concentration of UrKl/Cr in dogs with CKD2 was lower (8.46 pg/gCr) than in all groups of healthy dogs (GS: 13.00/BS: 8.78/SD: 10.75 pg/gCr) and in the group with CKD1 (13.61 pg/gCr). Concentration of UrKl/Cr was significantly lower in dogs with CKD2 (Figure 4) than in the control group (*p* = 0.0135). The reference interval (RI) for UrKl/Cr in all healthy dogs (*n* = 69) was determined to be 3.94–23.55 pg/gCr, and only in GS (*n* = 57) the RI was 3.85–24.62 pg/gCr. In the group of healthy GS, five outliers were identified and removed before determining the RI. No outliers were detected in the other groups of healthy dogs.

## 4 Discussion

In our study, we focused on the concentration of urinary soluble alpha klotho in healthy dogs and dogs with early stage CKD. The alpha-klotho protein sequence is known to be 88% identical between humans and dogs and 98% identical between humans and mice (29). In present study, the ELISA kit measuring canine-specific soluble alpha klotho was used. The use of an ELISA kit manufactured specifically for canine tests was expected to increase the accuracy of the test. Additionally, urine samples were never thawed before ELISA assay, as recent studies have shown that the performance of the same test is affected due to repeated cycles of freezing and thawing both clinical urine and serum samples (30, 31). On the contrary, in the study by Adema et al. (32), the concentration of soluble alpha klotho in freshly voided urine was significantly higher than in stored samples, indicating that klotho is unstable in stored human urine. To obtain accurate results, standardization should be developed in terms of sample processing, storage conditions, time point of analysis and assay kits used (31, 33). A previous human study evaluated the quality of three commonly used commercial ELISAs, among which there was considerable heterogeneity with within-run variations ranging from 4 to 32%, indicating the uncertainty of ELISA assays (33). Another report demonstrated that soluble klotho value by time-resolved fluorescence immunoassay (TRF) was associated with eGFR and soluble klotho value by ELISA was associated with age instead of eGFR (34). A recent study compared ELISA and immunoprecipitation immunoblot (IP-IB), where the level klotho determined by IP-IB showed a strong correlation with eGFR, but minimal correlation with ELISA (31). Despite the better performance of the TRF and IP-IB tests, the ELISA test is usually preferred because the kit is faster and cheaper in clinical practice.

In a study by Yi et al. (28), klotho in urine was significantly decreased in dogs with advanced CKD and correlated with various parameters, in contrast to serum alpha-klotho, which did not show these parameters correlations. This indicates that urinary klotho is more closely related to kidney function or damage then serum klotho. Also, in the study by Akimoto et al. (24), with human CKD patients, suggesting that the amount of klotho in urine, rather than klotho in serum, is related to the extent of nephron function. In this study, the amount of klotho excreted in urine per 24 h tended to decrease with the advancing stage of CKD, unlike the serum klotho level. Urinary klotho has potential as a biomarker of kidney disease or its progression, due to differences between stages of CKD disease and correlations with other parameters indicating kidney disease in previous studies. Moreover, the advantage of using this diagnostic or monitoring marker is a non-invasive intervention in the organism.

Expression of klotho genes is known to be age-related. A decrease in the concentration of soluble alpha klotho was observed in elderly people (35). In the study by Espuch-Oliver et al. (36) investigated serum soluble klotho reference values in a larger sample of 346 healthy adults also using ELISA. They observed that klotho levels varied significantly with age, and klotho was inversely correlated with age in healthy subjects. On the contrary, in the study by Yi et al. (28), which dealt with the level of soluble alpha klotho in the serum and urine of dogs, there were no significant differences between groups divided by age. In present study, the levels of soluble alpha klotho in urine were significantly higher in the group of older dogs over 6 years of age, while the lowest levels were in the group of dogs from 2 to 6 years. In a study of bonobos and chimpanzees, alpha klotho levels decreased with age, suggesting that this marker may have potential for studying the aging process at individual, population, species, and comparative levels. Regarding sex, female bonobos had higher levels of alpha klotho compared to males, and in chimpanzees, males had higher levels of alpha klotho than females, contradicting gender differences in life expectancy (37). In present study, no significant differences in the levels of soluble alpha klotho in urine between males and females in healthy dogs were observed. Gender differences in klotho concentration have not been confirmed in humans either (36).

Serum phosphate levels and phosphate toxicity show extremely strong negative correlations with lifespan in mammals (38–40). In our study, phosphorus levels were higher in small breed dogs and in German shepherds up to 2 years of age outside the reference range. Hyperphosphatemia is a physiological condition in growing dogs, when the need for phosphorus for bone development is increased. On the other hand, in aging dogs, hyperphosphatemia is most often associated with CKD. DNA damage in vascular smooth muscle cells caused by hyperphosphatemia leads to cellular senescence, which further contributes to the premature aging process seen with klotho dysregulation or loss (41, 42). Phosphorus levels were within the reference limit in our patients in the early stage of the disease. Previous studies suggest that klotho function and dysregulation of the FGF23-klotho pathway, causing hyperphosphatemia and endothelial dysfunction, are associated with the pathogenic mechanisms of CKD development and disease progression (21, 22, 27). In patients with progressive CKD, phosphate retention, hyperphosphatemia, higher levels of FGF-23 and low expression of klotho are observed. Many studies with animal and human CKD patients have shown decreased levels of alpha klotho, which further directly decreased with declining estimated glomerular filtration rate (eGFR) (21, 25, 27, 43–50). Soluble alpha klotho concentration may function as an early marker of CKD disease as it may reflect a decline in eGFR. Positive correlation between declining klotho levels and decline in eGFR has been observed in previous studies (47, 51–54).

A study by Yi et al. (28) investigated the characteristics of both serum and urinary klotho in dogs with CKD. UrKl/Cr in dogs with stage 3 and stage 4 of CKD was significantly lower than in dogs with stage 1 or stage 2 of CKD. In the CKD stage 3 group, UrKl/Cr was significantly lower compared to the control group and CKD stage 1 group. No significant differences were found in the CKD stage 4 group compared to the control group, although the median UrKl/Cr was lower than in the early stages of the disease. In our study, we mainly focused on UrKL/Cr concentrations in dogs with the early stages of CKD. The median UrKl/Cr in dogs in the CKD1 group was higher than in the control group, so we did not obtain any significant results. In the group of dogs with CKD2, the median UrKl/Cr was lower than in dogs with CKD1 and significantly lower than in healthy dogs. Since the total number of dogs with CKD in our study was lower, further studies are needed to investigate urinary alpha klotho as a marker in the early stages of this disease.

In addition, UrKl/Cr did not correlate with the tested parameters, namely SDMA, creatinine, urea and phosphorus levels, in healthy dogs, nor in dogs with the early stage of CKD. Unlike the previous study (28), where UrKl/Cr was inversely correlated with specific parameters, stepwise multiple regression analysis in this study showed that SDMA was an independent predictor of UrKl/Cr. Nevertheless, we are inclined to believe that the lower UrKl/Cr in CKD dogs and its correlation with kidney function parameters suggest that urinary alpha klotho is associated with CKD and disease progression. However, the low number of samples, the low quality of clinical studies, the lack of standardized tests and the lack of consensus on the processing of samples, especially in urine, explain the controversy regarding the applicability of soluble alpha klotho as a marker of CKD (55). Adequate standardization of new biomarker tests in dogs is necessary for reliable validation of results (56).

Friedrichs et al. (57) recommends a minimum of 120 reference individuals in order to determine reference limits by nonparametric methods with a 90% confidence intervals (CI). Reference intervals determined from smaller sample sizes are common and often necessary in veterinary medicine. However, the smaller the sample size, the higher the degree of uncertainty in estimating the reference limits. In our study, the number of samples was less than 120, but in study by Tassini et al. (58), a minimum number of observations was set at 50. Our study has several other limitations. In the group of healthy dogs, there were mainly individuals of one breed, namely the German Shepherd, an unequal number of males and females and unequal representation by age. However, we made the division into age groups based on statistical analysis. By testing one specific breed, we wanted to avoid at least breed differences, the influence of breeding and diet, since the dogs from our study are raised in the same conditions, fed the same food and all are active at work. Another limitation is the low number of dogs in the representation of the Belgian Shepherd breed, and in the representation of small and medium-sized dogs. For this reason, results in differences between breeds may not be statistically significant and can be further investigated. A few dogs in the healthy group had higher creatinine values according to the reference range in our laboratory (Crea < 88 μmol/L), but on the basis of the remaining laboratory, clinical and imaging examinations, they were classified as healthy dogs. The increased creatinine levels can be attributed to age, relatively young and old dogs, or exercise (59).

## 5 Conclusion

Alpha klotho was increased in the urine of aging dogs older than 6 years, but sex had no effect on its level. UrKl/Cr levels were decreased in dogs with CKD stage 2. Thus, it is possible that alpha klotho is related to CKD and its detection, although further research is needed to confirm its usefulness. The reference interval for healthy dogs was 3.94–23.55 pg/gCr, but alpha klotho values determined in dogs in the CKD1 and CKD2 groups are within this interval. For this purpose, further studies with a higher number of dogs and with standardization of the method and validation of the obtained results are needed.

## Data availability statement

The original contributions presented in the study are included in the article/supplementary material, further inquiries can be directed to the corresponding author.

## Ethics statement

Ethical approval was not required for the studies involving animals in accordance with the local legislation and institutional requirements because the University of Veterinary Medicine and Pharmacy in Košice does not require approval by the Ethics Committee regarding routine screening tests as part of the clinical activity. We performed non-invasive examinations and routine preventive sampling with the informed consent of the owners about the use of all tested parameters and animal data for the purpose of this publication. Written informed consent was obtained from the owners for the participation of their animals in this study.

## Author contributions

NM: Conceptualization, Data curation, Investigation, Methodology, Resources, Writing – original draft. JK: Data curation, Methodology, Writing – review & editing. CT: Data curation, Methodology, Writing – review & editing. AM: Funding acquisition, Investigation, Project administration, Writing – review & editing. MM: Investigation, Writing – review & editing. JF: Project administration, Writing – review & editing. SH: Supervision, Validation, Writing – review & editing.

## Footnotes

^1^http://www.iris-kidney.com/guidelines/staging.html
